# Automated Identification and Segmentation of *Ellipsoid Zone At-Risk* Using Deep Learning on SD-OCT for Predicting Progression in Dry AMD

**DOI:** 10.3390/diagnostics13061178

**Published:** 2023-03-20

**Authors:** Gagan Kalra, Hasan Cetin, Jon Whitney, Sari Yordi, Yavuz Cakir, Conor McConville, Victoria Whitmore, Michelle Bonnay, Jamie L. Reese, Sunil K. Srivastava, Justis P. Ehlers

**Affiliations:** Tony and Leona Campane Center for Excellence in Image-Guided Surgery and Advanced Imaging Research, Cole Eye Institute, Cleveland Clinic, Cleveland, OH 44195, USA

**Keywords:** ellipsoid zone integrity, photoreceptor damage, age-related macular degeneration, automated feature segmentation, deep learning, quantitative optical coherence tomography, geographic atrophy, progression prediction, clinical trial selection

## Abstract

Background: The development and testing of a deep learning (DL)-based approach for detection and measurement of regions of *Ellipsoid Zone* (EZ) *At-Risk* to study progression in nonexudative age-related macular degeneration (AMD). Methods: Used in DL model training and testing were 341 subjects with nonexudative AMD with or without geographic atrophy (GA). An independent dataset of 120 subjects were used for testing model performance for prediction of GA progression. Accuracy, specificity, sensitivity, and intraclass correlation coefficient (ICC) for DL-based *EZ At-Risk* percentage area measurement was calculated. Random forest-based feature ranking of *EZ At-Risk* was compared to previously validated quantitative OCT-based biomarkers. Results: The model achieved a detection accuracy of 99% (sensitivity = 99%; specificity = 100%) for *EZ At-Risk*. Automatic *EZ At-Risk* measurement achieved an accuracy of 90% (sensitivity = 90%; specificity = 84%) and the ICC compared to ground truth was high (0.83). In the independent dataset, higher baseline mean *EZ At-Risk* correlated with higher progression to GA at year 5 (*p* < 0.001). *EZ At-Risk* was a top ranked feature in the random forest assessment for GA prediction. Conclusions: This report describes a novel high performance DL-based model for the detection and measurement of *EZ At-Risk*. This biomarker showed promising results in predicting progression in nonexudative AMD patients.

## 1. Introduction

Geographic atrophy (GA) is a late-stage finding in age-related macular degeneration (AMD) that results in atrophic changes in the outer retinal layers and retinal pigment epithelium (RPE). The progression to GA in AMD and resultant permanent vision loss currently affects approximately 8 million people aged 55 years or older in the United States [1]. Increased prevalence of GA in AMD is seen with increasing age and in those with European descent [2]. With improved life expectancy and an aging population, the prevalence of GA and AMD disease burden are likely to increase further in the future [3].

The progression from AMD to GA is irreversible but stochastic. This necessitates screening and monitoring in all patients with nonexudative AMD, which can be resource intensive. This motivates a search for predictive features of AMD and GA progression. Previously carried out analyses of the natural history of AMD have identified qualitative signs in early and intermediate AMD that show predictive potential in the progression of GA. These features include hyperreflective foci and drusen morphology [4,5,6,7]. Recently, the Classification of Atrophy Meetings (CAM) consensus included photoreceptor damage in the recent definitions of incomplete RPE and Outer Retinal Atrophy (iRORA). iRORA is an early indicator of disease progression and was defined as a cluster of optical coherence tomography (OCT)-based features. This cluster of features, as noted by CAM consensus definitions, are (i) the presence of hyper-transmission into the choroid, (ii) partial RPE attenuation or disruption, and (iii) evident photoreceptor loss [8].

The ellipsoid zone (EZ) is a hyperreflective band visualized in the outer retina on OCT that is constituted by the mitochondria of the outer part of the photoreceptor inner segments. The reflectance of EZ is a likely indicator of photoreceptor mitochondrial health [9]. Additionally, OCT-based measurements of EZ integrity and sub-RPE compartment metrics have high importance in predicting disease progression in nonexudative AMD and GA [4,10,11,12,13,14,15]. Specifically, EZ integrity loss adjacent to GA margins has shown an association with GA progression [12,14,16,17,18,19]. This has prompted research that investigates the spatiotemporal connection between GA expansion and regions of EZ loss [12,14,16,17,18,19]. Without advanced image analysis systems, evaluating large datasets necessitates time-consuming and expensive manual feature annotation on OCT and fundus autofluorescence images [20]. These annotations are subject to inconsistency due to human error, especially with large longitudinal datasets [20]. With several clinical trials investigating novel treatments for nonexudative AMD as well as the recent FDA approval of the first treatment for GA in AMD (i.e., pegcetacoplan), exploration of photoreceptor damage denoted by EZ loss as a biomarker for profiling progression is of great interest.

Deep learning (DL)-based automated quantitative feature segmentation has addressed this challenge in clinical trials investigating a variety of pathologies and normative datasets [12,18,19,21,22,23,24]. Specifically, automated EZ segmentation using DL has shown great promise as an efficient and reproducible system for detecting and quantifying EZ integrity based on presence, distance from the RPE, or reflectivity [12,18,19]. However, there are no previously described DL systems that can automatically identify and measure areas of EZ specifically targeting at-risk areas for disease progression, such as GA development. This study describes a fully automated approach for the detection and pixel-wise segmentation of EZ regions at-risk of progressing to GA (*EZ At-Risk*) and/or degenerative changes using a DL-based method at the level of individual SD-OCT B-scans using a modified U-net architecture with more than 20 million parameters. Further, the measured percentage area of *EZ At-Risk* was tested as a potential biomarker for the prediction of GA progression in an independent dataset of nonexudative AMD patients.

## 2. Materials and Methods

All patient data included in this institutional review board-approved study was de-identified before use in compliance with Health Insurance Portability and Accountability Act regulations (HIPAA) guidelines. This study complied with the tenets of the Declaration of Helsinki. Given the retrospective nature of this assessment and as it de-identified imaging data, the requirement for informed consent was waived by the institutional review board.

### 2.1. Imaging and Data Collection

**Figure 1:**
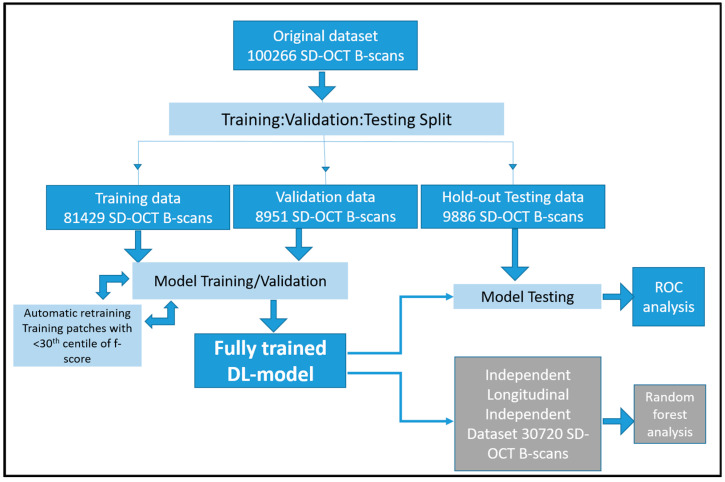
Flowchart of the data workflow during model training, validation, testing, and analysis. Independent random forest analysis was based on longitudinal dataset over a 5-year follow-up. The fully trained model was utilized in the DL-based measurement of percentage area of *EZ At-Risk*, which was subsequently tested in the random forest based predictive analysis of AMD progression. SD-OCT: spectral domain optical coherence tomography; DL: deep learning; EZ: ellipsoid zone; ROC: receiver operator curve.

A total of 100,266 SD-OCT B-scans from 900 visits of 341 de-identified patients with nonexudative AMD with or without GA were utilized in model building and testing. The workflow and data-size at each stage of the process is summarized in Figure 1. These images were captured using one of two devices, a Heidelberg Spectralis HRA + OCT (Heidelberg Engineering, Heidelberg, Germany) or Cirrus HD-OCT (Zeiss, Oberkochen, Germany). The OCT images were acquired with a 6 × 6 mm macular volume cube raster protocol with 97 or 49 B-scans for the Spectralis device and 128 B-scans for the Cirrus device. This combined dataset from different manufacturers was used to simulate a real world scenario and prevent model brittleness due to lack of diversity in the dataset source.

An independent dataset comprising 30,720 SD-OCT B-scans from 5-year follow-up of 120 de-identified patients (Figure 1) with nonexudative AMD with or without GA was included for the testing of *EZ At-Risk* as an independent biomarker in predictive analytics of GA progression. Sub-foveal GA (sfGA) in this dataset was defined as GA lesions that encroach on the foveal B-scan itself. Fovea-threatening involvement was defined as GA lesions that encroach on the central subfield (i.e., a fovea-centered circular region with a radius of 0.5 mm). Quantitative OCT-based biomarkers were defined as parameters that were measured on SD-OCT in a quantitative manner (such as a count of hyper reflective foci), in contrast to qualitative biomarkers (such as presence or absence of hyper reflective foci). Quantitative features from these images were obtained from a previously validated semi-automated DL-based approach [12,18,19,22]. These features included mean central subfield RPE–Bruchs membrane (BM) thickness (mean distance between the segmented RPE and BM layers on OCT B-scan), percentage area of partial EZ attenuation (percentage area of regions with distance between segmented EZ and RPE lines of <20 um on OCT B-scan), mean central subfield EZ–RPE thickness (mean distance between segmented EZ and RPE lines on OCT B-scan), percentage area of total EZ–RPE attenuation (percentage area of regions with distance between segmented EZ and RPE lines of 0um on OCT B-scan), and percentage area of RPE–BM attenuation (percentage area of regions with distance between segmented RPE and BM lines of 0um on OCT B-scan).

### 2.2. Ground Truth Retinal Layer Segmentation

All OCT B-scans were first segmented using a previously validated DL-enabled automatic multi-layer segmentation platform for the following layers: EZ, Bruch’s membrane (BM), and RPE [21,22,25,26]. The segmented scans were rigorously corrected by trained, expert readers followed by an independent review by a senior expert image analyst to ensure high quality and consistency in the ground truth. Any discrepancies arising in the first two layers of review were reconciled with a retina specialist. This process comprised the previously validated triple-layer expert review for generation of ground truth [21,22,25,26].

GA on OCT was defined as a region’s outer retinal atrophy on the B-scan where there was the complete absence of EZ and RPE. This is denoted by the presence of hypertransmission and associated retinal atrophy, based on the CAM consensus definitions [8].

*EZ At-Risk* training masks were defined as regions of ellipsoid zone attenuation that excluded regions that have already progressed to GA. *EZ At-Risk* was defined as the occurrence of EZ_RPE thinning of ≤10 um excluding any areas of GA.

### 2.3. Ellipsoid Zone At-Risk Detection Model

**Figure 2:**
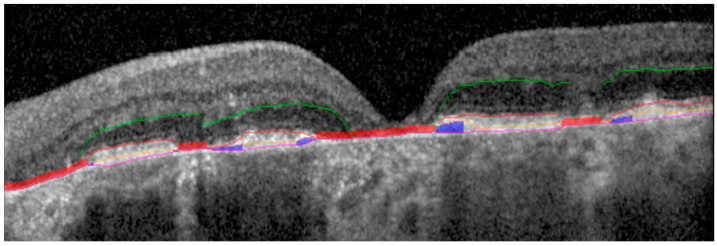
OCT B-scan showing multi-layer segmentation. From top to bottom: ONL (green line); EZ (red line); RPE (orange line); BM (pink line). Regions of GA (red overlay); regions of *EZ At-Risk* (blue overlay). OCT: optical coherence tomography; ONL: outer nuclear layer; EZ: ellipsoid zone; RPE: retinal pigment epithelium; BM: Bruchs membrane; GA: geographic atrophy.

Binary segmentation masks for each retinal layer of interest (EZ, RPE, and BM) were exported for individual B-scans, and areas of *EZ At-Risk* were defined as regions of EZ attenuation (EZ-RPE thickness ≤ 10 um), excluding the areas of GA (as defined by confluence of EZ, RPE, and BM lines based on segmentation protocols). This allowed for the identification of well-defined retinal layer segmentation-based regions constituting the ground truth masks for DL model training, based on the assumption that these areas reflect pre-GA areas of outer retinal attenuation. These masks were dilated to a thickness of 10 pixels centered on the EZ segmentation line to achieve contextual mask enrichment from adjacent contiguous regions (Figure 2).

### 2.4. Training and Validation Data

Eighty percent of all patients that were included in the study were randomly segregated into the training set (Figure 1). Visits from each patient with multiple visits were clustered together such that these data points only occurred in one dataset. This ensured the clear demarcation of datasets. The *EZ At-Risk* masks and corresponding original SD-OCT B-scans were used as training inputs for the DL model. Ten percent of all patients that were included in the study were randomly segregated into the validation set. This dataset was used to iteratively assess and improve performance during different epochs of model training.

### 2.5. Testing Data

Ten percent of all patients were randomly segregated in a hold-out test set (Figure 1). This dataset was curated while ensuring that no visits from a patient in this dataset were used for model training. This previously unseen or hold-out dataset was then deployed for performance assessment of the fully trained model. Model performance was also compared between imaging obtained using the two different imaging device types.

### 2.6. Deep Learning Architecture

A DL model was trained using a previously described UNet architecture with approximately 20 million parameters and 41 layers [27]. The architecture used images resized to 256 × 256 pixel as training inputs, kernel width of 5, early training stopping after 7 epochs without validation improvement, a batch size of 40, and samples per epoch of 200. The original OCT B-scans and DL outputs had matching scaling factors. This eliminated affection of the initial details of the original image. Additionally, the DL measurement was initially calculated with a very high precision (10,000th decimal place) to minimize errors in measurement due to resizing. A binary cross-entropy loss function and root mean squared optimizer was utilized in model training with a learning rate of 1 × 10^−4^.

### 2.7. Automatic Retraining

After the initial round of training, a sample of 100 patches was randomly selected from the training set and profiled for their F-scores, such that the 30th percentile of F-scores was identified (Figure 1). The patches with F-scores lower than the 30th percentile were duplicated within the training set and a second round of training was conducted using the same model and parameters. This was used as a data augmentation method that allowed fine-tuning of the model on training examples with poor performance.

### 2.8. Statistical Analysis

For the performance assessment of the DL model, the entire hold-out test set was evaluated for accuracy, specificity, and sensitivity for obtaining pixel-wise *EZ At-Risk* percentage area measurement. The receiver operator curve (ROC) was plotted for the DL model and the area-under-curve (AUC) was calculated to assess performance. Additionally, intraclass correlation (ICC) coefficient was calculated to compare the DL model output and ground truth measurements of the *EZ At-Risk* area.

In an independent longitudinal dataset of 120 patients, the percentage of the *EZ At-Risk* area was assessed for correlation against growth in the GA area. Further, a random forest model with 10-fold cross validation was created in this independent dataset to predict progression to sub-foveal GA development using percentage area of *EZ At-Risk*, mean central subfield RPE–BM thickness, percentage area of partial EZ attenuation, mean central subfield EZ–RPE thickness, percentage area of total EZ–RPE attenuation, and percentage area of RPE–BM attenuation. Ranked feature importance was assessed for the *EZ At-Risk* area compared to other quantitative OCT-based biomarkers. The ROC was plotted for the random forest model and the AUC was calculated to assess performance. Pearson’s correlation was used to assess correlation of the *EZ At-Risk* area and growth in the GA area. A two-sample two-sided t-test was used to compare the mean *EZ At-Risk* area between eyes that showed growth in the GA area and those that did not show growth in the GA area. Statistical significance was assumed at *p* < 0.05.

The model training, statistical analysis, and data visualizations were performed using Python (v3.9.11) and R (v4.0.1, Bell Laboratories, Murray Hill, NJ, USA). Model training was carried out locally using workstations with an Intel Xeon processor (10 cores, 20 threads), dual NVIDIA RTX 2080-Ti graphics processing unit setup (12 gigabytes of VRAM each), and 128 gigabytes of system memory.

## 3. Results

### 3.1. DL-Based Automated Detection and Measurement of Regions with EZ At-Risk

Binary detection of the presence of *EZ At-Risk* at the level of the entire OCT volume using the fully automated DL model achieved an accuracy of 99% with a sensitivity of 99% and a specificity of 100%. This binary detection performance at the level of an individual OCT B-scan achieved an accuracy of 87% with a sensitivity of 96% and a specificity of 73%.

**Figure 3:**
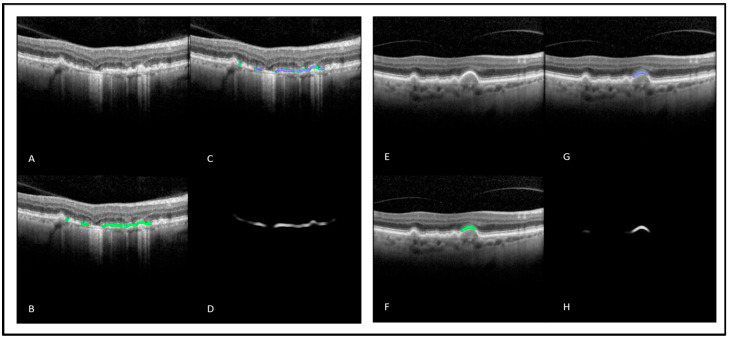
Output of the DL-based *EZ At-Risk* detection and segmentation model. (**A**,**E**) raw OCT B-scan; (**B**,**F**) OCT B-scan with ground truth *EZ At-Risk* mask overlay (green); (**C**,**G**) OCT B-scan with true positive DL output overlay (blue), false negative DL output overlay (green), false positive DL output overlay (orange); (**D**,**H**) DL gray scale output. DL: deep learning; EZ: ellipsoid zone; OCT: optical coherence tomography.

**Figure 4:**
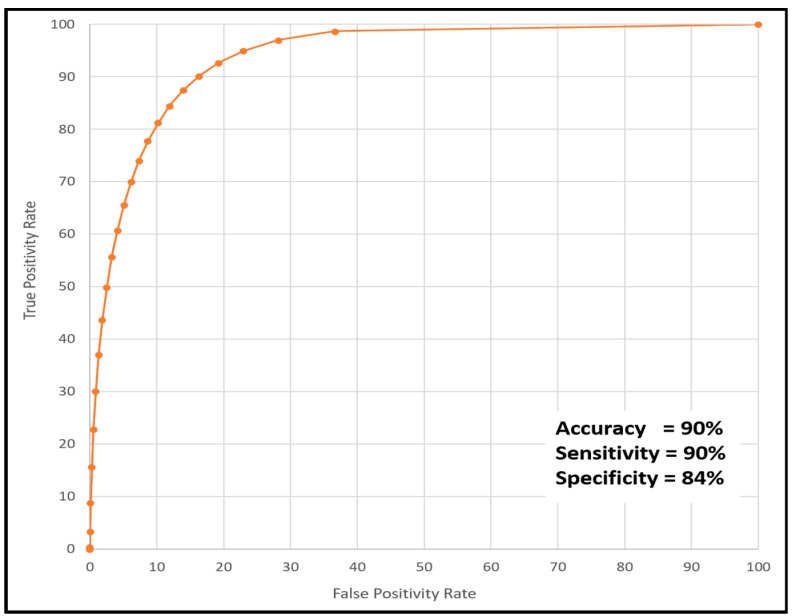
ROC illustration of the DL-based *EZ At-Risk* segmentation model at the individual SD-OCT B-scan level with accuracy, sensitivity, and specificity. ROC: receiver operator curve; DL: deep learning: EZ: ellipsoid zone; SD-OCT: spectral domain optical coherence tomography.

Automated measurement of *EZ At-Risk* percentage area at the level of the individual OCT-B scan achieved an AUC of 97%, an accuracy of 90%, a sensitivity of 90%, and a specificity of 84%. The model output on the OCT B-scans can be visualized in Figure 3. The ROC visualizing the model performance across different thresholds is illustrated in Figure 4. In the subset analysis assessing model performance for the two device types, comparable performance was achieved with both devices (accuracy: 78% vs. 83%) with slightly higher performance with the Spectralis device. The percentage area of regions with *EZ At-Risk* automatically detected using the DL-based model showed an ICC of 0.83 (*p* < 0.001) when compared with the ground truth annotation. These results are summarized in Table 1.

### 3.2. Correlation with Growth of GA Lesions

In the independent assessment of the longitudinal follow-up of patients with nonexudative AMD, percentage area of automatically detected *EZ At-Risk* showed significant positive association with the growth of geographic atrophy at year 5 of follow-up (*p* < 0.001). The eyes that showed an increase in geographic atrophy lesion area showed a significantly higher (*p* < 0.001) mean percentage area of *EZ At-Risk* at the baseline (7.3%) compared to eyes that did not show any increase in GA lesion area (2.3%) at year 5 of follow-up (*p* = 0.001). Eyes that showed conversion to sub-foveal GA or threatening foveal involvement had higher *EZ At-Risk* at the baseline (7.8%) compared to eyes that showed no conversion or fovea-threatening involvement (1.8%) at 5 years of follow-up (*p* < 0.001).

### 3.3. Random Forest Prediction of Sub-Foveal GA

**Figure 5:**
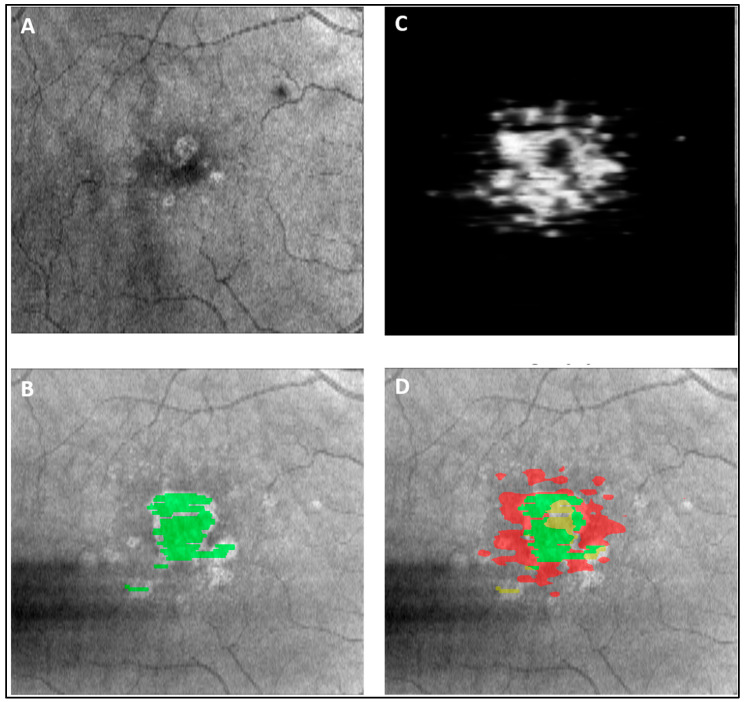
A case example of GA progression on en face OCT from (**A**) baseline scan to (**B**) 5-year follow-up scan (GA area labeled in green). (**C**) en face compilation of DL grayscale output of *EZ At-Risk* measurement on baseline OCT. (**D**) overlay of baseline DL output and year 5 follow-up GA ground truth showcasing the prediction potential of baseline *EZ At-Risk* measurement where green region indicates successful prediction of GA at year 5, red indicates false positive GA prediction for year 5, and yellow indicates false negative GA prediction at year 5. GA: geographic atrophy; OCT: optical coherence tomography; DL: deep learning; EZ: ellipsoid zone.

**Figure 6:**
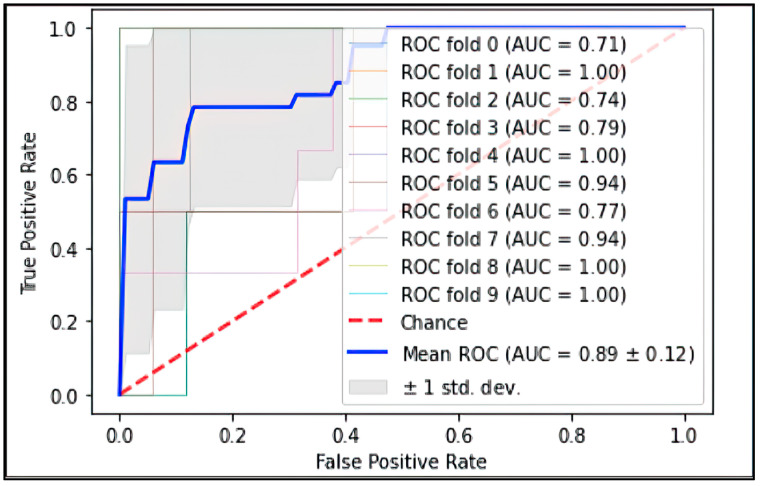
ROC illustration of the random forest model for prediction of sfGA using quantitative OCT-biomarkers including *EZ At-Risk*. Ten-fold cross validation was performed, and mean ROC results are represented by the blue solid line while random chance is represented by the red dashed line. ROC: receiver operator curve; sfGA: sub-foveal geographic atrophy; OCT: optical coherence tomography; EZ: ellipsoid zone.

The random forest generated with 10-fold cross-validation using only the higher-order OCT features achieved an AUC of 0.90 with a sensitivity of 80% and a specificity of 90% for prediction of conversion to sfGA or fovea-threatening involvement. The model features that showed the highest importance in achieving this classification were percentage area with total EZ attenuation, percentage area of *EZ At-Risk*, percentage area of partial EZ attenuation, baseline EZ–RPE central subfield thickness (in µm), baseline mean RPE–BM central subfield thickness (in µm), and percentage area of complete RPE atrophy. A representative case showcasing the utility of *EZ At-Risk* measured at the baseline in predicting sfGA or fovea-threatening involvement at year 5 is shown in Figure 5. The ROC curve for the random forest classifier is depicted in Figure 6.

## 4. Discussion

This report demonstrates a DL-enabled high-performance automated model for the detection of regions of *EZ At-Risk* (accuracy = 99%, sensitivity = 99%, specificity = 100%) in patients with nonexudative AMD using OCT B-scans from different device types and pixel-accurate measurement of these regions (AUC = 97%, accuracy = 90%, sensitivity = 90%, specificity = 84%). Further, the utility of *EZ At-Risk* as an automatically measured GA biomarker was studied by assessing correlations between the percentage area of *EZ At-Risk* at baseline with growth in the GA area in an independent longitudinal cohort of nonexudative AMD patients over 5 years (R = 0.48, *p* < 0.001) (Figure 1). Finally, the ranked feature importance of this biomarker in predicting progression to sfGA and fovea-threatening involvement was shown to be one of the highest using a random forest classifier that comprised additional previously reported higher-order OCT features [12].

This report demonstrates the utility of DL-enabled fully automated approaches for quantifying *EZ At-Risk* regions in nonexudative AMD patients. This approach can detect EZ integrity loss, representing photoreceptor damage. Previous attempts to measure EZ integrity loss in other pathologies include an automated detection of EZ integrity loss related to trauma. This pipeline had an accuracy of 85% with a sensitivity of 85% and specificity of 85% [28]. Another automatic DL-based quantification algorithm was used to detect EZ loss in 85 hydroxychloroquine retinopathy patients with an overall accuracy of 90% [29]. Similarly, automated EZ integrity loss detection in mild diabetic retinopathy achieved an accuracy of 90% in a small cohort of 13 patients [30]. A recent method utilized 40 OCT volumes from 40 patients with diabetic macular edema and retinal vein occlusion to develop an ensemble-based approach to achieve accurate segmentation and quantification of the photoreceptor layer [31]. This method was then utilized to automatically obtain photoreceptor thickness maps to assess GA progression in the FILLY clinical trial with 57 eyes over a 12-month follow-up [32]. Authors described utilization of the local progression rate in an attempt to holistically capture disease progression, as opposed to merely assessing global disease burden [32]. The current report, by comparison, describes a model that is trained on a much larger and diverse dataset comprising 341 patients with dry AMD for training and testing, followed by an independent longitudinal cohort of 120 eyes with a 5-year follow-up, which should significantly improve performance generalizability. Additionally, the current report achieves a highly accurate segmentation of EZ regions at-risk for progression to GA. This is in contrast to the previously described method that only achieves segmentation of the overall photoreceptor layer and requires additional thickness map analysis of potential regions at risk of progression [31,32]. Therefore, this novel approach described in the current report is higher-order in comparison to previously described methods. Even though a direct comparison is not possible between the methods due to the nature of differences between the segmentation outputs, the AUC of previously described ensemble approach was 96% [31], which is lower in comparison to the currently described method (AUC = 97%). Additionally, the current method retained this high performance across different device types (i.e., Cirrus and Spectralis devices), whereas the previous reports are limited to only one device type [33,34]. The pixel-wise segmentation accuracy of *EZ At-Risk* regions of 90% described in the current report is in line with the previous reports from other diseases [29,30,31]. Another important distinction from previously reported methods is the exclusion of regions with EZ loss in areas of pre-existing GA from the model training and analysis. This allowed for the measurement of *EZ At-Risk*—an improved biomarker targeting areas of de novo development of GA and potential expansion of existing GA lesions.

Recent studies have used EZ integrity loss and sub-RPE quantification to assess GA expansion [4,10,11,12,13,14]. In an analysis of 30 eyes, 13 eyes were shown to have preceding outer retinal disruption and EZ loss on OCT imaging that progressed to GA [16]. In another report of 49 patients, authors described that sub-RPE drusenoid deposits and associated EZ loss were associated with the progression of GA [15]. In a previous report from 29 patients, authors described a predictive GA model that utilized an automated feature extraction pipeline. Even though that study’s results might not be generalizable due to small sample size, loss of EZ integrity was one of its top features for predicting GA growth [10]. In an analysis of 137 patients, authors described sub-RPE compartment changes and EZ integrity loss that allowed for the creation of an automated predictive model of sub-foveal GA progression [12]. A randomized clinical trial of risuteganib therapy in 39 subjects showed improvement in EZ integrity with associated improvement in visual acuity and slowed progression of GA [18]. Recently, independent randomized clinical trials of avacincaptad pegol and elamipretide have shown slower progression of GA in eyes that had reduction in EZ integrity loss [33,34]. The current report builds on these previously reported findings by utilizing a large training dataset for creation of a fully automated DL-model that can automatically measure areas of *EZ At-Risk* in areas where GA is not present. This measurement was used in an independent random forest analysis of 120 eyes based only on quantitative OCT features for prediction of progression to subfoveal GA and fovea-threatening involvement. In this analysis, the percentage of *EZ At-Risk* area was identified as one of the most significant features for predicting GA progression.

Quantification of *EZ At-Risk* has clinical applications in predictive modeling and prognostication of nonexudative AMD and GA [4,10,11,12,13,14]. Additionally, automatically generated metrics could profile patients in large scale clinical trials. Patients with high levels of *EZ At-Risk* may be at higher risk for progression and selected for enrollment, resulting in higher yield, and reducing the number needed to treat.

This study has some important limitations that should be acknowledged. The data and analysis came from a single, quaternary-level academic institution and the results derived from this study may not be generalizable to other populations or sites. Future work is currently underway that explores the applicability of this model in a large dataset from other clinical sites. In addition, this study did not thoroughly examine the variations in U-Net architecture to find the most optimal architecture for *EZ At-Risk* detection and segmentation as the purpose of this analysis was to establish novelty and proof of concept for *EZ At-Risk* itself. A more complex neural architecture may have improved model performance, and this is currently being tested for future applications of this model. Finally, the random forest feature ranked importance analysis was based on a relatively small sample size of 120 patients. Additionally, the absence of OCTs between follow-up intervals makes it challenging to determine the exact timing of sfGA conversion. Efforts are currently underway to validate these findings in a large external dataset that can allow for the standardization of the prediction time interval as well.

However, this study utilized a large dataset for deep learning, using a previously validated semi-automated multi-layer retinal layer segmentation approach. The model performance could be generalized well across imaging devices tested. Further, percent area of *EZ At-Risk* area was tested as a biomarker for GA progression in an independent longitudinal dataset with 5 years of follow-up; it ranked highly in random forest-based feature importance in predicting sfGA and fovea-threatening involvement conversion. This allowed for a direct comparison between *EZ At-Risk* area and previously reported higher-order SD-OCT quantitative features shown to predict sfGA and fovea-threatening involvement conversion.

## 5. Conclusions

This study demonstrates a DL-based approach capable of automatic detection and measurement of regions with *EZ At-Risk* in SD-OCT B-scans. The percentage area of *EZ At-Risk* was shown to be one of the highest ranked features in predicting progression and conversion to sub-foveal GA in an independent dataset. Ongoing and future efforts in this area include independent validation of these findings in a larger external dataset.

## Figures and Tables

**Table 1:** Summary of DL-based results for detection and measurement of *EZ At-Risk*. DL: deep learning; EZ: ellipsoid zone.

Parameter	Value
**Detection performance at the level of overall OCT volume**	
Accuracy	99%
Sensitivity	99%
Specificity	100%
**Detection performance at the level of individual OCT-B scans**	
Accuracy	87%
Sensitivity	96%
Specificity	73%
**Measurement performance at the level of individual OCT-B scans**	
AUC	97%
Accuracy	90%
Sensitivity	90%
Specificity	84%
ICC	0.83 (*p* < 0.001)

## Data Availability

Not applicable.
